# Preparation of an MHC Alloy by Direct Doping of HfC and Its High Temperature Oxidation and Volatilization Behavior

**DOI:** 10.3390/ma15051958

**Published:** 2022-03-07

**Authors:** Miao Wang, Shuangping Yang, Jie Dong, Haixing Sun, Shouman Liu

**Affiliations:** 1 School of Metallurgical Engineering, Xi’an University of Architecture and Technology, Xi’an 710055, China; dongjiexyz@xauat.edu.cn (J.D.); shx20220110@xauat.edu.cn (H.S.); liusm617100@xauat.edu.cn (S.L.); 2 Shaanxi Province Metallurgical Engineering and Technology Research Center, Xi’an University of Architecture and Technology, Xi’an 710055, China

**Keywords:** molybdenum alloy, MHC alloy, high energy ball milling, oxidation, oxidation weight loss, volatilization, MoO_3_, powder metallurgy

## Abstract

An HfC-doped molybdenum (Mo-Hf-C; MHC) alloy was prepared via a powder metallurgy process, including dry direct doping followed by ball-milling, cold-isotactic-pressing, and vacuum sintering. An oxidation comparison experiment was conducted, and the oxidation and volatilization behaviors were analyzed using the mass change, volatile generation rate, and morphology transformation. The results show that relatively uniform powder morphology can be obtained by the direct doping of carbide and high-energy ball milling. The oxidation of the MHC alloy at a lower temperature was characterized by the oxygen-absorption and a slight weight gain, while at a higher temperature and longer holding time, it was characterized by the mass volatile weight loss. A significant weight change appeared at 800 °C for 30 min with a weight loss rate of 4.8%. Surface oxidation products developed horizontally from ridged oxides at lower temperature stages to a flaky oxide layer at higher temperatures. The peeling of the oxide layer was the result of interfacial pore development, which led to exposure of the alloy matrix and further oxidation. Based on the oxidation and volatilization characteristics of HfC-doped MHC alloys, we conclude that the oxidation and volatilization of the MHC alloy conformed to the general law; however, the significant weight loss temperature, weight loss rate, volatilization temperature, and volatilization rate were improved compared with pure molybdenum and traditional molybdenum alloys, thus, indicating that the precipitation of the second phase HfC particles at the grain boundaries and within the grains can inhibit the oxidation and volatilization of matrix elements to a certain extent.

## 1. Introduction

The high melting point refractory metal molybdenum and its alloys have attracted extensive attention in the fields of aerospace, machinery, and metallurgy; the chemical industry; nuclear industry; and so on. However, the application of molybdenum and its alloys at high temperature is limited by the oxidation resistance, which has not been solved. 

Under normal temperatures, the metal molybdenum can exist stably; however, when heated to about 300 °C in air, it begins to oxidize, and a cyan oxide film forms; at higher than 600 °C, a tightly adhered dark green oxide layer is formed; and, at even higher temperatures, the oxidation is accelerated, and the formed MoO_3_ with high vapor pressure causes a large amount of volatilization causing serious weight loss of the metal molybdenum [1,2]. In addition, with the Mo:O mole ration of n:3n-1 under air or water vapor oxidizing conditions at 615–780 °C, a series of unstable intermediate oxides can be formed, and continuous oxidation will lead to the conversion of unstable oxides to MoO_3_ and further volatilization at high temperatures. 

It can be seen that the main characters of metal molybdenum oxidation are a wide oxidation reaction temperature range, various types of oxidation products, poor stability of most oxidation products, and high temperature volatility of stable oxidation products [1,2]. Therefore, the main reason attribute to the poor high temperature oxidation resistance is that, with the increase of the holding time and oxidation temperature, unstable intermediate oxides will be converted into low-melting and volatile MoO_3_, which cannot prevent further oxidation of the matrix metal but will cause catastrophic damage due to oxide peeling and volatilization instead.

The destructive oxidation of molybdenum at high temperature severely limits its application as a heat-resistant structural material. Under high temperature conditions, compared with the degradation of its own performance, the anti-oxidative corrosion ability of the material surface, which can be improved by coating and alloying, often plays a more significant role in the long-term service [3]. Among them, alloying is one of the most common and effective methods [4].

Kunming Pan et al. [5] reviewed the high temperature oxidation behavior of a Mo-Si-B alloy, indicating that micro-alloying, microstructure size, oxidizing atmosphere, Si/B atomic ratio, etc. all affected the oxidation resistance. The research results of Bin Li et al. [6] showed that the oxidation resistance of an Mo-Si-B alloy at 1300 °C were improved by doping of TiO_2W_, the function of which is divided into two parts, dissolving in SiO_2_ phase to improve the fluidity of the alloy in the initial oxidation stage by the formation of a continuous distributing oxide layer and preventing the volatilization of MoO_3_ and the internal diffusion of oxygen in the stable oxidation stage by the formation of borosilicate and the formation of a large number of ZrO_2_/ZrSiO_4_ particles. 

Wei-qiang Hu et al. [7,8] prepared Y_2_O_3_ and TiC strengthened W alloys using a freeze-drying process. A nano-scale uniform distributed second phase within the grain and grain boundaries was obtained, thus, providing new idea for the toughening and toughening preparation of refractory metals. Yu Zhitao et al. [9] found that TZM alloys were extremely easy to oxidize first at the grain boundary and at defects, followed by eroding into the matrix. A high oxygen content increased the possibility of the transformation of metal carbides into oxides and thus weakened the dispersion strengthen effect; therefore, low oxygen preparation technology is an important direction to improve the oxidation resistance. 

Kuai-she Wang et al. [10,11] prepared La-TZM alloy sheets and studied their oxidation behavior, indicating that the tensile strength and elongation were significantly improved compared to traditional TZM alloys, and the formation of a dense oxide layer effectively prevented the transfer of oxygen due to the pinning effect of the fine La_2_O_3_ second phase at the grain boundary. Patrice et al. [12] proposed that the strengthening and toughening of polycrystalline alloys at high temperature could be achieved by HfC doping, and the microstructure of the alloy was composed of a dendritic austenite matrix and eutectic carbides, which are beneficial to high temperature strength. 

Yong Wei et al. [13] prepared CNTs/HfH_2_-strengthened HfC-doped molybdenum (Mo-Hf-C; MHC) alloys by a powder metallurgy process. Through dispersion strengthening, grain refinement, lattice distortion, and purification of free oxygen, the relative density, microstructure, and mechanical properties were improved. Yang Lilin et al. [14] studied the ablation properties using a plasma ablation system of tungsten-molybdenum alloy doping with different HfC contents. The results showed that HfC existed at a stagnation point due to its high melting point, and thus a small amount of HfO_2_ was formed covering the surface of the substrate and improving the ablation performance of the alloy.

It can be seen that, due to the doping of different elements and the preparation process, the oxidation and high-temperature volatilization weight loss behavior of certain alloy will be different from that of pure molybdenum and other types of molybdenum alloys. The physicochemical properties and volatilization characteristics of the oxidation corrosion products determine whether the alloy continues to oxidize or the severity of oxidation and the course of oxidation corrosion after the initial oxidation. 

Therefore, it is necessary to explore and master the morphology and properties of specific molybdenum alloy oxidation products as well as the volatilization crystallization law caused by further oxidation. In this study, FCC HfC, which has the highest melting point and optimal thermodynamic stability among all carbides, was adopted to strengthen the molybdenum matrix by dry-directly introducing the powder metallurgy process to prepare an MHC (Mo-Hf-C) alloy. 

The Vickers hardness was 46.5% higher than that of pure molybdenum prepared by the same PM process. By performing high temperature oxidation experiments, combined with XRD, SEM, and EDS analysis, the possible physical and chemical reactions, product transformation, and morphological changes of MHC alloys under different oxidation conditions were explored. Furthermore, the oxidation corrosion mechanism and second phase anti-corrosion mechanism were analyzed, providing a theoretical and practical basis for improving the anti-oxidation ability of molybdenum alloys and their performance at high temperatures for the further development and application of MHC alloys.

## 2. Materials and Methods

### 2.1. Alloy Preparation and Oxidation Experiment

The preparation process of the MHC alloy included alloy powder batching → vacuum drying → mixing →cold isostatic compaction → vacuum sintering → rotary forging. The purity of the reduced Mo powder (Jinduicheng Molybdenum Co. Ltd., Xi’an, China) and HfC powder (Tianjin Guangfu fine chemical research institute, Tianjin, China) are all greater than 99.5%.

**Mixing:** After weighing (with HfC of 1.2 wt.%), blending, vacuum drying, and 160-mesh pre-screening, powder mixing was conducted in an omnidirectional planetary ball mill (DQM-4L, Chunlong instrument, Lianyungang, China), adopting agate balls as an abrasive medium with balls 3:2 (Chunlong instrument, Lianyungang, China). The powder ball milling adopted the forward and reverse rotation process, and the total abrasive mixing time was 5.5 h.

**Powder compaction:** The mixed powder as pressed by a cold isostatic press (Deyang Xihai isostatic pressure equipment manufacturing Co. Ltd., Deyang, China) with compaction strength of 180 MPa in a 25 mm hose for 10 min. The primary and secondary pressure relief times were 90 and 15 s, respectively, producing a green compact with a diameter of 20 mm.

**Green compact sintering and rotary forging:** The compact was placed in a medium frequency vacuum induction sintering furnace (with vacuum degree of 6.7 × 10^−2^ Pa and sintering pressure of 35 Mpa, Hengjin Vacuum, Shenyang, China). Five temperature stages were set for segmented sintering, which were 800, 1200, 1500, 1750, and 1950 °C, and 2 h sintering at each temperature stage was kept, respectively, with a maximum sintering temperature of 0.8 Tm (Tm is the melting point of Mo). The staged sintering process can remove moisture, oxygen, adsorbed gas, organic matter, and internal stress of the green compact in the early stage of heating; remove low-melting-point impurities and hydrogen generated from the powder in the middle stage of sintering; and finally, the green compact is sintered into a molybdenum alloy billet product with dense metallic properties. The relative density of billet was higher than 98% by the Archimedes drainage method [15]. The sintered billet was subjected to rotary forging (Baoding Yanxing machinery manufacturing Co. Ltd., Baoding, China) and straightening treatment (Shakewei automation equipment, Wuxi, China) at 1200 °C with a pass deformation rate of 15% to obtain a bar with a diameter of 12 mm.

**Figure 1:**
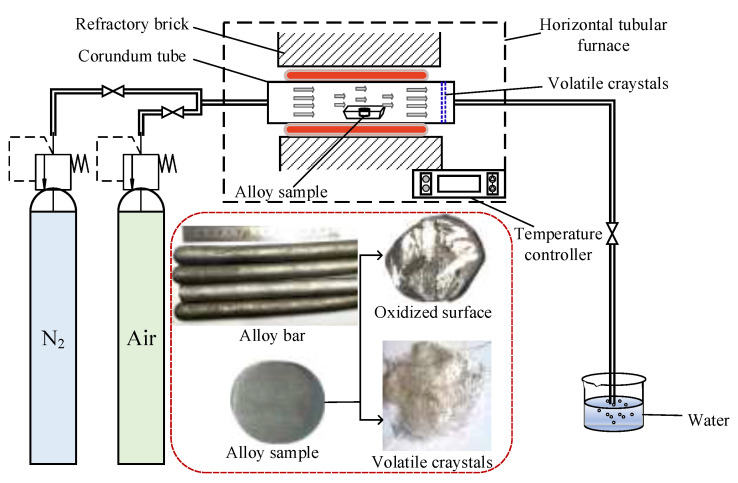
Schematic diagram of the experiment setup.

**Sample preparation and oxidation experiment:** The prepared bar was cut into ø10 × 5 mm specimens by a wire cutting process (Taizhou Weihai CNC machine tool Co. Ltd., Taizhou, China). The surface of the specimen was ground and polished with sandpaper (Jinshifeng abrasive product Co. Ltd., Ganzhou, China), followed by ultrasonic cleaning with ethanol and vacuum drying of samples (Binglin, Shanghai, China). The oxidation experiment was conducted in a high temperature tube furnace (NBD-T1700, Henan nobody materials science and technology Co. Ltd., Zhengzhou, China), and a corundum boat was used to hold the sample and placed in the high temperature zone of the tube furnace as shown in Figure 1. The sample was protected by 400 mL/min of N_2_ from room temperature to the target oxidation temperature and the cooling process and was oxidized by standard air at target temperature. The arrows in Figure 1 indicate the direction of airflow, and the blue marked position at the end of the corundum tube indicates the crystalline area where the oxidized volatiles were collected.

### 2.2. Characterization of Oxidation Products and Volatiles

**XRD analysis:** X-ray diffraction (XRD) is an effective means to study the mineral phase composition and microcrystalline structure. According to the X-ray diffraction analysis results of MHC alloys under different oxidation conditions, the phase composition and microcrystalline structure of the oxidation products can be obtained. The XRD detection of the samples was performed using a D8 ADVANCE X-ray diffractometer from BRUKER, Karlsruhe, Germany. The main working parameters were: copper target, tube current, and voltage of 40 and 40 mA, respectively. The scanning step width and step length were 0.01° and 0.05°, respectively. The 2θ scan rage was 15–90°.

**SEM analysis:** Scanning electron microscopy (SEM) is an effective means to observe the three-dimensional morphology of materials and analyze the composition of micro-areas. The morphological characteristics, such as crystal morphology, grain size, surface defects, and whiskers of the oxidized surface and volatile can be observed by SEM (TESCAN VEGA II, Brno, Czech).

## 3. Results and Discussion

### 3.1. Preparation of Alloy Powder by High-Energy Ball Milling

**Figure 2:**
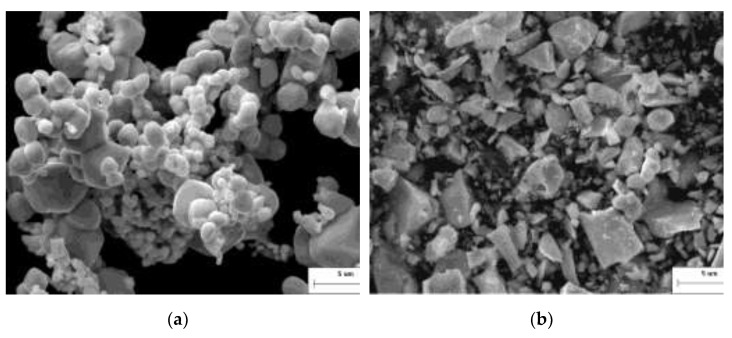
Morphology of the raw material alloy powder. (**a**) Raw Mo powder and (**b**) HfC powder.

**Figure 3:**
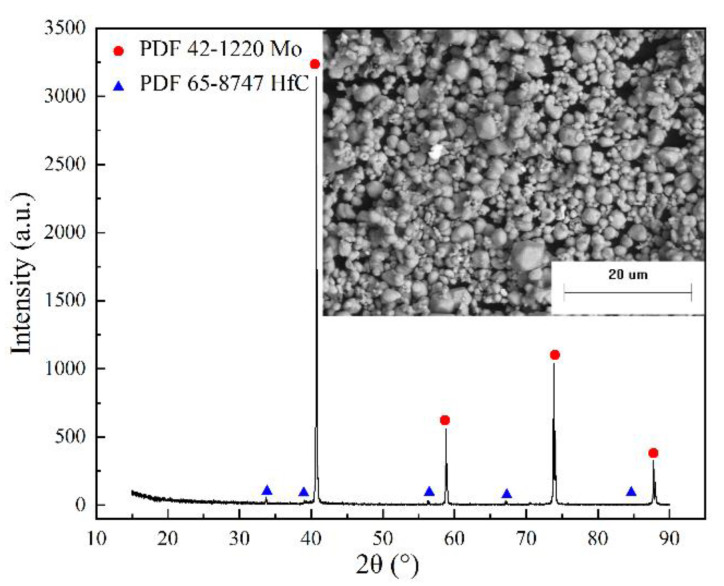
XRD and SEM analysis of the alloy powder.

Mo powder and mixed powder formed by mixing process are the key raw materials for the production of MHC alloys. Chemical reactions, structure, and property changes are induced by mechanical energy provided by high-energy ball milling process. The blending powder is subjected to repeated strong impacts and rolling and shearing between the grinding ball and the powder material, resulting in fractures and evenly mixing during ball milling—the most commonly used mixing method. In this study, high-energy ball milling was adopted to prepare and mix the MHC alloy powder. The morphology of the raw Mo powder and HfC powder is shown in Figure 2. The XRD and SEM analysis of the MHC alloy powder after batching and ball milling for 5.5 h are shown in Figure 3.

It can be seen from Figure 2 that the raw Mo powder (Figure 2a) was nearly spherical with clear particle boundaries, while the raw HfC powder (Figure 2b) was a brittle block polyhedral structure with good particle dispersion, whose particle size was close to that of raw Mo powder. A chemical reaction between raw mixing powders cannot be induced by the high-energy ball milling from Figure 3 where the matrix phase and the doped second phase remained in the original form. 

**Figure 4:**
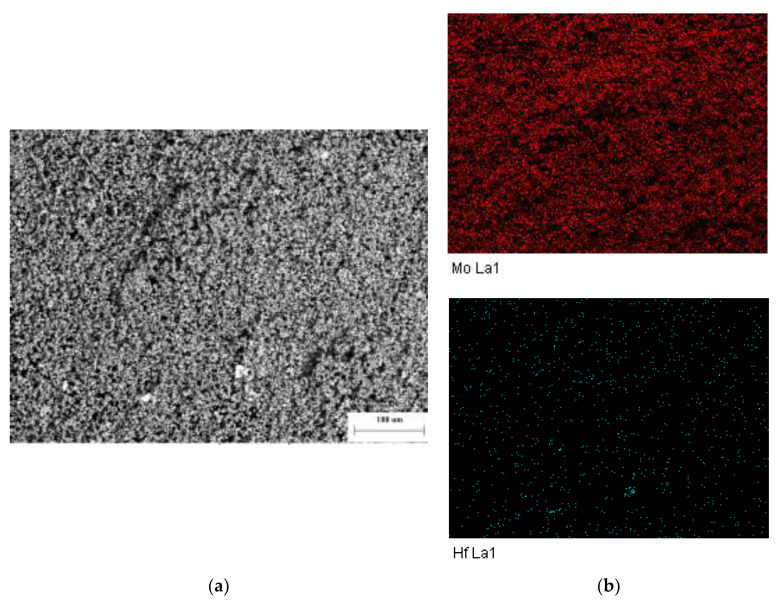
Low magnification SEM and element surface scanning analysis of the MHC alloy powder. (**a**) Low magnification SEM of the alloy mixed powder. (**b**) Surface scanning of Mo and Hf.

A uniform morphology of the alloy powder was prepared without fusion and adhesion between particles, which was beneficial to obtain a compact and uniform structure in the following pressing process. In order to evaluate the effect of the ball milling process on the uniform dispersion of the second phase in the matrix, surface scans of Mo and Hf elements were performed on randomly selected parts of the MHC alloy powder prepared by ball milling at low magnification as shown in Figure 4.

The spot shown in Figure 4 indicates the positions of the particles. It can be seen that the surface scan distribution of the Mo element shows larger red particles compared with the Hf element, which are both uniformly dispersed without agglomeration. The distribution position of the Hf element is similar to that of the Mo element, indicating that the fine Mo matrix and HfC particles were initially adhered together with each other. The existing dry direct doping ball milling process can obtain an alloy powder with a fine particle size, uniform distribution of second phase elements, and uniform morphology, thus, fulfilling the powder preparation requirements.

### 3.2. Weight Loss Behavior and Oxide Formation Characteristics

**Figure 5:**
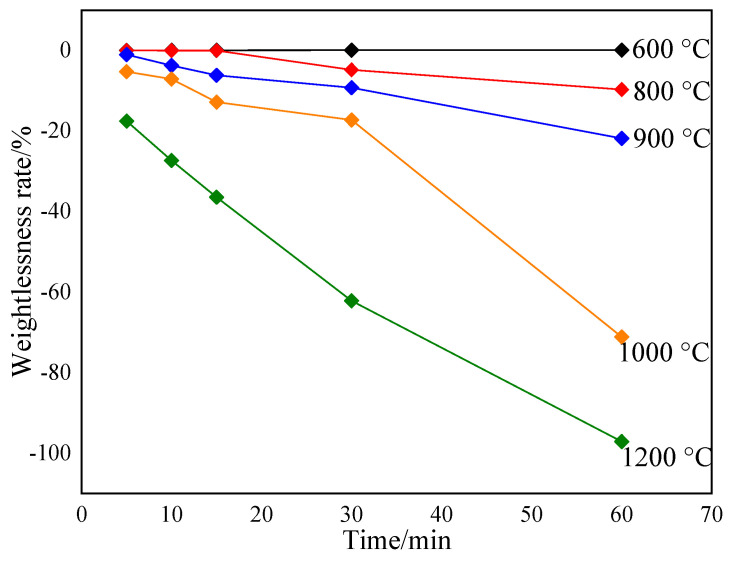
Weight change rates under different oxidation temperatures and holding times.

Oxidation experiments were conducted on MHC alloy bars at predetermined temperatures for different time periods, and the weight change is shown in Figure 5. It can be seen that the oxidation of the MHC alloy showed a similar trend to that of pure Mo—that is, the weight loss rate increased with the prolongation of oxidation time or the increase of oxidation temperature. However, the temperatures at which slight weight loss and significant weight loss occurs of the MHC alloy were higher than those of pure molybdenum, reported TZM alloys and La-TZM alloys [10].

The alloy samples continued to lose weight with the prolongation of the oxidation time and the increase of the oxidation temperature from Figure 5. Different from the high temperature weight loss, a slight weight gain of 0.003–0.092% was observed when oxidized at 600 °C for 5–60 min and oxidized at 800 °C for 5 min, indicating that the oxidation process at lower temperature or shorter holding time only developed to slightly weight increase by oxygen absorption, indicating that the alloy can be applied to working condition of 600 °C for a long time or 800 °C for a short time without destructive oxidation corrosion compared with pure Mo starts to oxidize at 300 °C. 

The obvious weight change condition for the MHC alloy was at 800 °C for 30 min, with a weight loss rate of 4.8%, which increased to 14.325% when holding for 60 min. As shown in Figure 5, the weight change rate and oxidation behavior progressed slowly when oxidizing for 5 and 15 min under the experimental conditions, while severe weight loss occurred when oxidized for 30 and 60 min. In addition, when the alloy was exposed to a high-temperature oxidizing atmosphere above 1000 °C for a long time, the heat released by the oxidation reaction could not be dissipated in time, and the autogenous temperature increased beyond the melting point of MoO_3_, resulting in an autocatalytic effect of the oxidation process [1]. 

**Figure 6:**
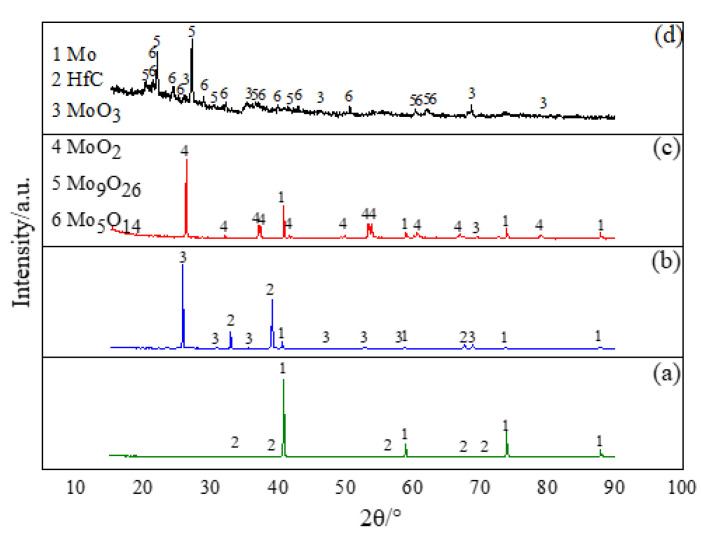
XRD patterns of the sample surface under different oxidation conditions. (**a**) MHC-HfC; (**b**) 800 °C—15 min; (**c**) 1000 °C—15 min; and (**d**) 1200 °C—15 min.

This eventually led to rapid volatizing of the oxidation product and catastrophic destruction of the alloy. Under the existing experimental conditions, the weight loss rates of oxidation at 1000 °C for 5, 15, 30, and 60 min were 7.374%, 12.816%, 17.264%, and 71.12%, respectively; the weight loss rates of oxidation at 1200 °C for 5, 15, 30, and 60 min were 17.516%, 27.32%, 62.14% and 97.09%, respectively. The oxidation reaction was accompanied by the volatilization of MoO_3_, by which more matrix was exposed to the oxidizing atmosphere, and further oxidizing reactions were induced. Figure 6 shows the surface XRD patterns of the MHC alloy under different oxidation conditions. The higher the temperature, the more serious the weight loss.

As can be seen from Figure 6b, the main phase of the alloy surface was MoO_3_. When the MHC alloy was oxidized at 800 °C for 15 min, it can be judged that the alloy was not oxidized to form volatile MoO_3_ or the formed MoO_3_ had not yet reached the volatile activity combining with the weight loss rate. After oxidation at 1000 °C for 15 min (from Figure 6c), the main oxides phase on the alloy surface changed to MoO_2_ due to the volatilization of large amount MoO_3_, indicating that the residual MoO_3_ was about to be volatilized, which will result in a large area of exposure of the metal matrix to the oxidizing atmosphere for further oxidation reaction. 

When the alloy was oxidized at 1200 °C for 15 min (from Figure 6d), it can be concluded that the increase of temperature promoted the transition of the initial oxide MoO_2_ to Mo_5_O_14_, Mo_9_O_26_, and other intermediate oxides. With the increase of oxygen absorption, the intermediate oxide was finally converted into MoO_3_, whose formation speed and volatilization speed were further accelerated with the increase of temperature and the prolongation of holding time. Therefore, the oxidation product phase of the MHC alloy was complicated at higher temperatures, including initial oxides and unstable transitions oxides.

### 3.3. Oxidative Volatilization Behavior of MHC Alloys

In further prolonging the oxidation time or increasing temperature, the oxides generated on the alloy surface volatilized into the gas phase, and continued to volatilize and flow with gas stream to the low temperature zone for crystallization until the partial pressure of the gas phase and the saturated vapor pressure were balanced when the partial pressure of the gas phase was less than the saturated vapor pressure at the corresponding temperature. 

**Figure 7:**
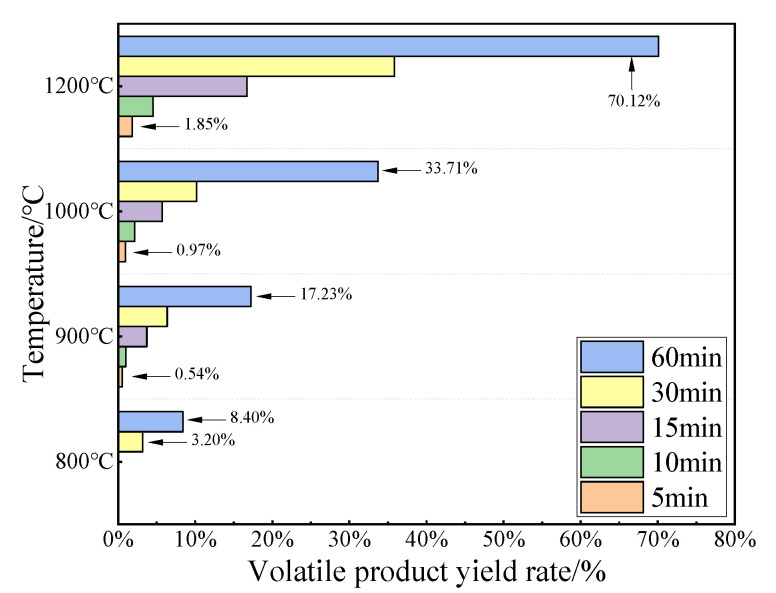
The formation rate of volatile product during MHC alloy oxidation.

**Figure 8:**
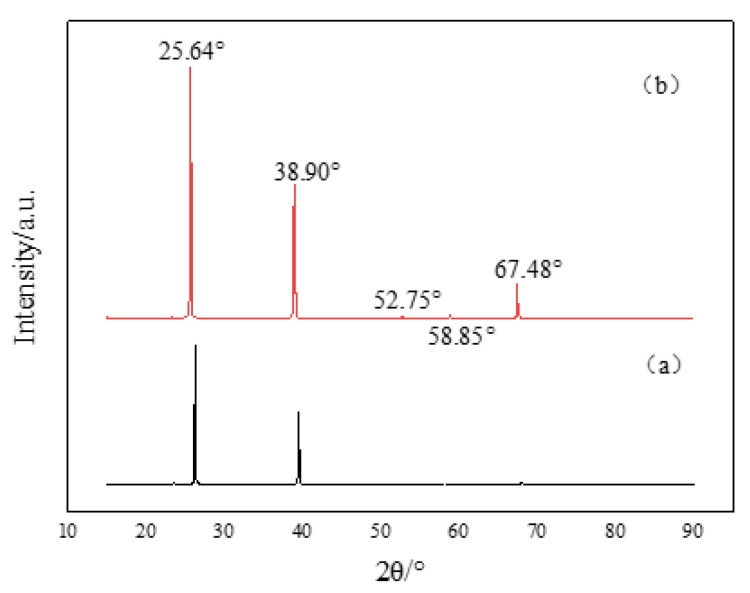
Macroscopic morphology and phase analysis of volatile crystals. (**a**) 1000 °C—15 min. (**b**) 1200 °C—1 h.

When the oxidation temperature rose above 800 °C, the MHC alloy began to lose weight significantly due to the massive volatilization of MoO_3_. Figure 7 shows the formation rate of volatiles during MHC alloy oxidation process. The macroscopic morphology and phase analysis of the volatile crystals under different oxidation conditions are shown in Figure 8. The study by Gulbransen and Jansson [16] indicated that volatility has an important effect on the high temperature oxidation of molybdenum.

It can be seen from Figure 7 that the volatiles generation rate trend is similar to that of weight change rate—that is, the volatilization rate increased with the prolongation of the oxidation reaction time or the increase of the oxidation temperature. When oxidized at 600 °C for 5–60 min and 800 °C for 5 min, no volatiles were formed, and the alloy was still in the stage of slow weight gain by oxygen absorption from previous conclusion. When oxidized at 800 °C for 30 min, weight loss occurred due to the significant volatilization of MoO_3_, with a volatile crystal generation rate of 3.20%. Further increasing the temperature to 900, 1000, and 1200 °C and holding for 5–60 min, the maximum generation rates of volatiles were 17.23%, 33.71% and 70.12%, respectively.

As can be seen from Figure 8, the phase composition of volatile crystals was high-purity MoO_3_ with different morphologies. With oxidation at 1000 °C for 15 min, transparent, slender mesh-like volatiles with a generation rate of 3.72% were collected, while with oxidation at 1200 °C for 60 min, light yellow-white, finely fragmented, and brittle volatiles with a generation rate of 70.12% were collected. The characteristics of the micro-morphology of the oxide volatile crystal were determined by the growth mechanism of the crystal. 

The MoO_3_ volatile crystal was developed centering on the crystal nucleus and growing by three-dimensional attachment of crystal particles, such as atoms, molecules, and ions, and the concentration difference between the crystal surrounding and the whole medium system iwass the driving force for crystal growth. In the process of the MHC alloy oxidation, the surface first absorbs oxygen to form unstable oxidation products Mo_4_O_11_, Mo_5_O_14_, Mo_6_O_17_, etc., which is further oxidized and transformed into stable MoO_3_ and MoO_2_ with the increase of temperature and the internal stress of the oxide layer, resulting in the continuous separation and volatilization MoO_3_ particles on the surface of the alloy and entering the gas flow. 

Therefore, during the movement of the gas flow to the low temperature region, some tiny and stable nucleus was formed by collision of particles, which become the core of subsequent crystallization. When the gas flow continuously transports the oxide gas phase to the crystal nucleus, the volatilization crystal will continue to grow.

### 3.4. Oxidation and Volatilization Mechanism of MHC Alloys

**Figure 9:**
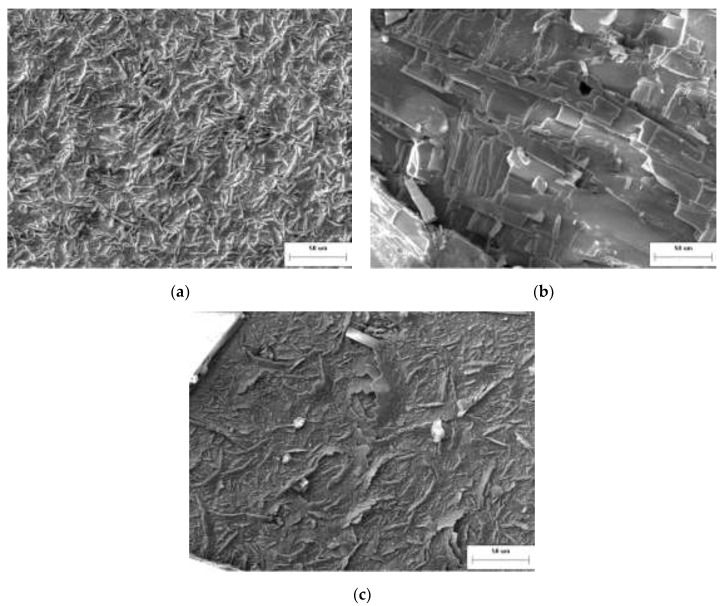
Surface morphology of the MHC alloy before and after oxidation. (**a**) 600 °C—30 min; (**b**) 800 °C—30 min; and (**c**) 1200 °C—5 min.

**Figure 10:**
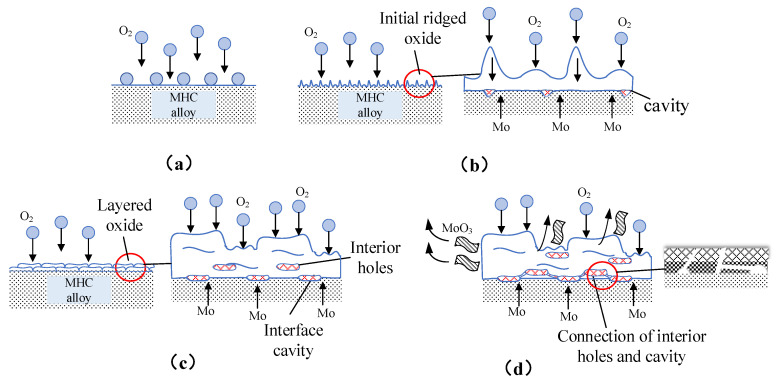
Schematic diagram of oxidation and volatilization mechanism of the MHC alloy. (**a**) Oxygen absorption; (**b**) formation of oxide ridges and interfacial cavities at low temperature; (**c**) formation of flaky oxide layer and the growth of internal pores; and (**d**) interconnection of internal pores, peeling, and volatilization of layered oxides.

The smooth surface of the alloy was covered with defects after oxidation. Figure 9 shows the surface morphology of the alloy oxidized at different condition, and Figure 10 is a schematic diagram for the development and mechanism of oxidation and volatilization process of the MHC alloy. The ridge-like oxide products were generated at low temperatures, while layered oxide products and internal pores appeared at higher temperatures.

The morphology of surface oxides were non-planar ridge-like when oxidation experiment was conducted at 600 °C for 30 min, due to the uneven oxygen adsorption and thus uneven volume change as shown in Figure 9a and Figure 10a,b. The growth of surface oxides was caused by the reverse diffusion and combination of Mo and O_2_ along the grain boundary of the oxide product film on the surface of the alloy [17]. 

The increase of the temperature and holding time resulted in the lateral growth of oxides, which developed into a layered form as shown in Figure 9b and Figure 10c. The surface oxide film grows by the mass transfer of Mo inside the grains, which is microscopically inhomogeneous, leaving Mo vacancies at the interface between the alloy matrix and the oxide products. When the vacancy concentration reaches super saturation, the vacancies gradually grow into the inner cavity and interior holes with a connection between them as shown in Figure 10b,c.

With the increase of temperature, necking was formed, reduced, and sealed into the oxide layer due to the squeeze of cavity by the formation and squeeze of layered oxides, forming interface cavity, which have a great influence on the adhesion of the oxidation products. As can be seen from Figure 10, when the interface cavity connects and develops into the non-contact area of the oxide product-alloy interface, the oxide film will be cracked and peeled off from the alloy matrix by internal stress, resulting in either a further exposure to the oxidizing atmosphere of the MHC alloy or lifting, bulging, and layered growth of oxide layers that have not yet been peeled off (Figure 9c).

This provides a larger specific surface area for volatilization transformation by further increasing the atmospheric oxygen partial pressure or temperature and holding time. Therefore, inhibiting the formation of the non-contact interface between the oxidation product and the alloy matrix during oxidation can effectively improve the adhesion and interface properties between both, thereby, improving the high temperature oxidation resistance of the alloy.

Non-volatile MoO_2_ is formed on the surface of a traditional TZM alloy with a slow oxidation process when oxidized at 400 °C and below, and a rapid weight increase occurs when oxidized at 400–750 °C, while significant volatilization of MoO_3_ occurs when oxidized at higher than 750 °C. As a comparison, the significant oxidation temperature of La-TZM alloy from the literature [10] was 600 °C, and the weight loss rate of oxidizing at 1000 °C for 30 min was 91.83%. The weight loss rate and volatile generation rate of the MHC alloy prepared in this study were 4.8% and 3.2%, respectively, when oxidized at 800 °C for 30 min. 

The weight loss rate of oxidation at 1000 °C for 30 min was 17.26%. The temperature at which the weight loss reaction begins was 50 °C higher than that of traditional TZM alloy, and the anti-oxidative weight loss performance of MHC was also improved than that of traditional TZM alloy and the La-TZM, indicating that the erosion of oxygen to the molybdenum matrix of the MHC alloy was effectively hindered.

**Figure 11:**
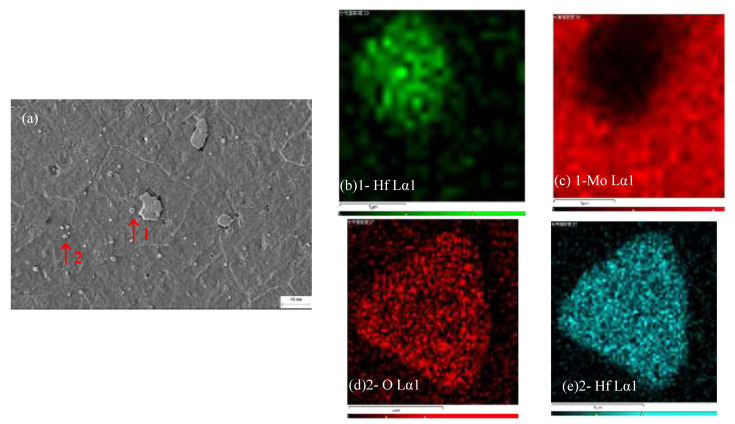
SEM and EDS analysis of the MHC alloy. (**a**) Microstructure of the MHC alloy; (**b**,**c**) Hf and Mo EDS analysis of precipitate 1; and (**d**,**e**) O and Hf EDS analysis of precipitate 2.

Figure 11a shows the microstructure of the MHC alloy, and EDS was performed on the precipitated particles as shown in Figure 11b–e.

It can be seen from the analysis results that Hf dispersed and precipitated on and within the grain boundaries of the alloy in the form of oxides or carbides, pinning at the grain boundaries, which prevented the dislocation activity of the alloy and refined the grains. The finer the grains of the alloy, the larger the volume fraction of the grain boundaries. Although the diffusion coefficient of oxygen in the grain boundaries was greater than the bulk diffusion coefficient [18], due to the precipitation of HfC in the grains and grain boundaries and its ultra-high melting point of HfC (3830 °C), the combination of Mo matrix and oxygen was hindered, preventing the dissolution and the migration of matrix elements [14]. 

Therefore, the weight loss rate was lower than that of pure molybdenum and the La-TZM [10]. In addition, the XRD results show that the volatile crystals ere pure MoO_3_ as the oxidation process and the volatile crystallization process of the MHC alloy were in a dynamic equilibrium at high temperature, proceeding and developing simultaneously; thus, the inhibition of oxidation, in turn, inhibited the volatilization of MoO_3_ to a certain extent.

## 4. Conclusions

(1) A relatively uniform morphology and second phase distribution alloy powder was obtained by dry direct doping of HfC powder into the Mo powder matrix followed by a high-energy ball milling process for 5.5 h.
(2) The prepared alloy can be applied at 600 °C and below without obvious weight changes, and the performance above 600 °C was also improved. The significant weight loss rate in the oxidation experiment for the MHC alloy was 4.8% when oxidized at 800 °C for 30 min. The existing alloying method can increase the violent volatilization temperature; therefore, research on the directional formation of protective oxide films during the oxidation process and the inhibition of the volatilization behavior of molybdenum oxides is essential for the design of molybdenum alloys.
(3) The oxidation process of MHC alloys consist of two stages: the oxygen absorption and weight gain at a lower temperature stage with a shorter holding time, where a ridged oxide was formed, and the slow oxygen absorption and a large amount of volatilization at higher temperature stage with a longer holding time, where the ridged oxide developed laterally into a layered or layered superimposed oxide film.
(4) The oxidation products of MHC alloys have limited protection ability on metal matrix, and the surface defects formed by the peeling of the oxides create conditions for the absorption of oxygen and volatilization. Therefore, inhibiting the interface cavity during oxidization is one of the favorable conditions for the enhancement of interface performance with a metal matrix and oxide product.
(5) The precipitation of HfC particles at the grain boundary and within the grain is conducive to inhibiting the oxidation and volatilization of the Mo element in the alloy; the high-temperature performance of molybdenum alloy is limited by the oxidation and volatilization characteristics; therefore, the concept of doping non-volatile second phase to inhibit of the volatilization of MoO_3_ or form a new non-volatile phase between the Mo matrix and second phase can be one of the directions for alloy design.

## Data Availability

Data is contained within the article.
